# Age and Diet Affect Gene Expression Profiles in Canine Liver Tissue

**DOI:** 10.1371/journal.pone.0013319

**Published:** 2010-10-12

**Authors:** Dong Yong Kil, Brittany M. Vester Boler, Carolyn J. Apanavicius, Lawrence B. Schook, Kelly S. Swanson

**Affiliations:** 1 Department of Animal Sciences, University of Illinois, Urbana, Illinois, United States of America; 2 Division of Nutritional Sciences, University of Illinois, Urbana, Illinois, United States of America; 3 Department of Veterinary Clinical Medicine, University of Illinois, Urbana, Illinois, United States of America; Instituto de Química - Universidade de São Paulo, Brazil

## Abstract

**Background:**

The liver plays a central role in nutrient and xenobiotic metabolism, but its functionality declines with age. Senior dogs suffer from many of the chronic hepatic diseases as elderly humans, with age-related alterations in liver function influenced by diet. However, a large-scale molecular analysis of the liver tissue as affected by age and diet has not been reported in dogs.

**Methodology/Principal Findings:**

Liver tissue samples were collected from six senior (12-year old) and six young adult (1-year old) female beagles fed an animal protein-based diet (APB) or a plant protein-based diet (PPB) for 12 months. Total RNA in the liver tissue was extracted and hybridized to Affymetrix GeneChip® Canine Genome Arrays. Using a 2.0-fold cutoff and false discovery rate <0.10, our results indicated that expression of 234 genes was altered by age, while 137 genes were differentially expressed by diet. Based on functional classification, genes affected by age and/or diet were involved in cellular development, nutrient metabolism, and signal transduction. In general, gene expression suggested that senior dogs had an increased risk of the progression of liver disease and dysfunction, as observed in aged humans and rodents. In particular for aged liver, genes related to inflammation, oxidative stress, and glycolysis were up-regulated, whereas genes related to regeneration, xenobiotic metabolism, and cholesterol trafficking were down-regulated. Diet-associated changes in gene expression were more common in young adult dogs (33 genes) as compared to senior dogs (3 genes).

**Conclusion:**

Our results provide molecular insight pertaining to the aged canine liver and its predisposition to disease and abnormalities. Therefore, our data may aid in future research pertaining to age-associated alterations in hepatic function or identification of potential targets for nutritional management as a means to decrease incidence of age-dependent liver dysfunction.

## Introduction

The liver is the central organ in the regulation of nutrient metabolism, xenobiotic metabolism, and detoxification. Evidence from humans and rodents has indicated that aging leads to a marked change in the liver structure and function [1]. In general, aged liver is characterized by a decline in weight, blood flow, regeneration rate, and detoxification, which have been related to an increased risk of liver abnormalities in the elderly [1]. Moreover, age-associated changes in liver function are expected to be affected by diet because dietary nutrient metabolism is centered in the liver. However, molecular mechanisms underlying the effects of age and diet on liver physiology and pathogenesis remain inconclusive.

Recent advances in microarray technology and bioinformatics allow the analysis of genome-wide gene expression changes, providing a useful link between complex molecular events and physiological responses by identifying specific genes and metabolic pathways involved [2]. Although senior dogs suffer from many of the chronic diseases present in the elderly [3], molecular analyses of canine tissues have been rarely performed. To our knowledge, no data pertaining to a large-scale molecular analysis of the liver tissue in senior vs. young adult dogs have been reported.

As a starting point, our laboratory performed an experiment designed to measure physiological response of healthy adult dogs as a function of age and diet [4]. Young adult or senior dogs were fed either an animal protein-based diet (APB) containing high dietary fat and low fiber or a plant protein-based diet (PPB) containing moderate fat and high fiber. We found that the diet altered nutrient digestibility, blood chemistry, gastrointestinal morphology, and microbial fermentation, with the effects being dependent on age [4], [5]. In previous publications, we reported the effects of age and diet on gene expression alterations in cerebral cortex [6], adipose tissue [7], and skeletal muscle [8] of the dogs that were used by Swanson et al. [4]. In the current experiment, total RNA was isolated from the liver tissue collected from our previous experiment [4], comparing hepatic gene expression profiles as a function of age and diet using commercial canine microarrays. Given the fact that the liver plays a central role in nutrient metabolism and its functionality declines with age, it was hypothesized that hepatic gene expression would be largely dysregulated in senior vs. young adult dogs and this impairment would be exacerbated by feeding APB diet containing animal-derived lipids high in saturated fatty acids and cholesterol.

## Results

**Figure 1:**
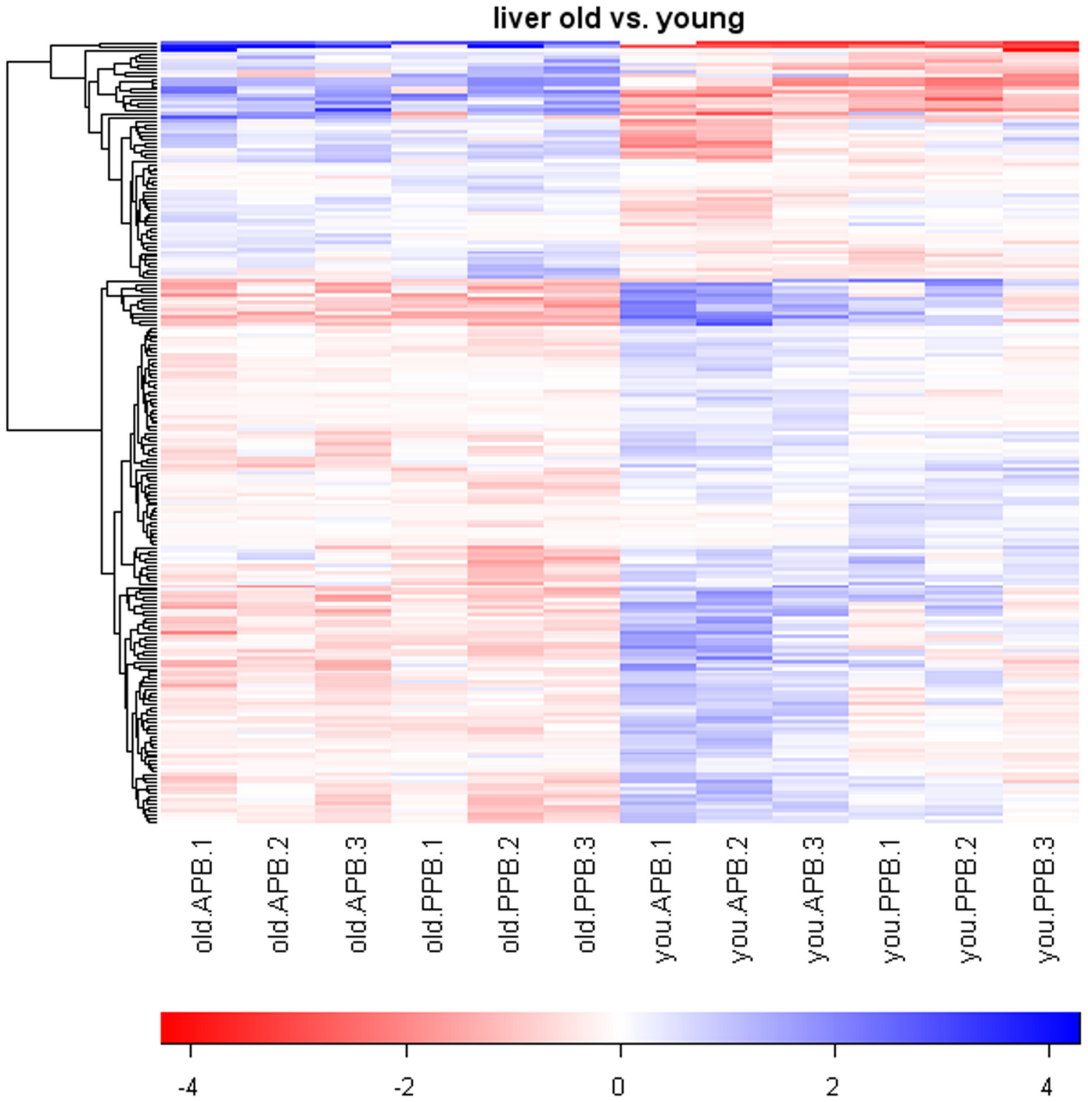
Heatmap of senior vs. young adult dog pairwise comparisons. Values are the GCRMA-processed probe set value (Log_2_ scale) minus the mean value for that probe set across all arrays. The dendrogram was created by hierarchical cluster analysis.

**Figure 2:**
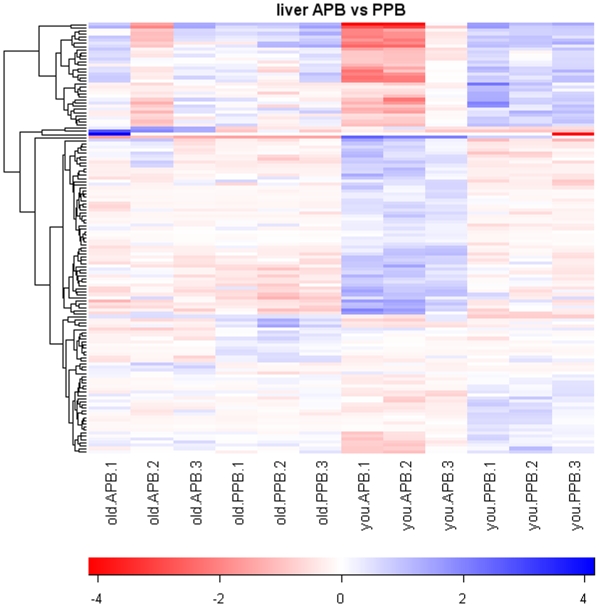
Heatmap of animal-protein based diet (APB) vs. plant-protein based diet (PPB) pairwise comparisons. Values are the GCRMA-processed probe set value (Log_2_ scale) minus the mean value for that probe set across all arrays. The dendrogram was created by hierarchical cluster analysis.

Based on a 2.0-fold change cutoff and FDR <0.10, a total of 371 gene transcripts were differentially expressed by age (234 genes) and/or diet (137 genes), according to the pre-planned statistical screening methods (Table 1). The heat map in Figure 1 indicates significant and consistent gene expression changes due to age, and within age groups dietary treatment had a greater impact on gene expression changes in young dogs than in senior dogs. However, when genes altered by diet were clustered, inconsistent pattern was observed (Figure 2). Following removal of unannotated genes and duplicate probe sets of the same gene, 89 genes were identified as being differentially expressed by age (53 genes) and/or diet (36 genes). Twenty five genes were up-regulated (Tables 2 and 3) and 28 genes were down-regulated (Tables 4 and 5) with increased age. Nine genes were up-regulated (Table 6) and 27 genes were down-regulated (Tables 7 and 8) in dogs fed the APB vs. PPB diet.

**Table 1:** Global view of liver gene expression alterations in senior vs. young adult dogs fed an animal protein-based diet (APB) or plant protein-based diet (PPB).

	Number of gene transcripts altered^1^	Number of annotated genes altered^2^
Total genes differentially expressed	371 (2.7%)	89
Age-associated alterations	234 (1.7%)	53
Up-regulated	76	25
Down-regulated	158	28
Diet-associated alterations	137 (1.0%)	36
Up-regulated	65	9
Down-regulated	72	27

1 Values in the parenthesis represent the percentage of gene transcripts differentially expressed in relation to the total number of genes expressed in the liver tissue (13,778 genes).

2 Number of annotated and non-redundant genes that had >2.0 fold-change in gene expression.

**Table 2:** Up-regulated cell growth and development-, cellular metabolism-, and cell signaling and signal transduction-associated genes in hepatic tissue of senior vs. young adult dogs fed an animal protein-based (APB) or plant protein-based (PPB) diet.

			Fold Change	
Functional classification	Gene name	Symbol	APB	PPB
**Cell growth and development**				
Tumor marker	WAP four-disulfide core domain 2	WFDC2	107.0	21.97
Cell adhesion	CD99 molecule	CD99	3.17	
p53 in cell cycle arrest	G-2 and S-phase expressed 1	GTSE1		2.24
**Cellular metabolism**				
Amino acid metabolism	D-amino-acid oxidase	DAO	2.67	
Carbohydrate metabolism	Phosphofructokinase	PFKP		4.27
Carbohydrate metabolism	6-phosphofructo-2-kinase/fructose-2,6-biphosphatase3	PFKFB3		3.10
Lipid metabolism	Fatty acid desaturase 3	FADS3	4.91	
Lipid metabolism	Retinol dehydrogenase 16	RDH16		2.38
Glycolipid synthetic process	Forssman glycolipid synthetase (FS)	GBGT1	3.59	4.49
Glyoxylate and dicarboxylate metabolism	Hydroxyacid oxidase 2	HAO2	2.12	
Xenobiotic metabolism (detoxification)	Glutathione S-transferase, pi 1	GSTP1	4.41	12.60
Xenobiotic metabolism	Carboxylesterase 2	CES2	3.87	
**Cell signaling and signal transduction**				
RAS signaling pathway	RAS guanyl releasing protein 1	RASGRP1		2.63

**Table 3:** Up-regulated cellular trafficking and protein processing-, immune and stress response-, and transcription-translation-associated genes in hepatic tissue of senior vs. young adult dogs fed an animal protein-based (APB) or plant protein-based (PPB) diet.

			Fold Change	
Functional classification	Gene name	Symbol	APB	PPB
**Cellular trafficking and protein processing**				
Transmembrane transport	Peroxisomal membrane protein 69	ABCD4	3.13	
Synaptic transmission	Synaptophysin-like 1	SYPL1	2.11	
Protein ubiquitination	Ubiquitin-like 1 activating enzyme E1A	SAE1		2.27
**Immune and stress response**				
Immune response	Galectin-2	LGALS2	2.07	
Immune response	Ig heavy chain V-III region VH26 precursor	LOC607467		9.66
**Transcription-translation**				
Transcription	Ribosomal protein L6	RPL6	3.53	
Telomere maintenance	Telomeric repeat binding factor 2 interacting protein 1	TERF2IP	2.73	
**Miscellaneous and unknown**				
Muscle growth	Musculoskeletal, embryonic nuclear protein 1	MUSTN1		64.66
Calcium channel activity	Glycoprotein M6A	GPM6A	8.73	8.32
Fibrinolysis	Annexin A2	ANXA2	7.82	
Unknown	FUN14 domain containing 2	FUNDC2		2.45
Unknown	Transmembrane 6 superfamily member 1	TM6SF1	3.82	

**Table 4:** Down-regulated cell growth and development-, cellular metabolism-, and cell signaling and signal transduction-associated genes in hepatic tissue of senior vs. young adult dogs fed an animal protein-based (APB) or plant protein-based (PPB) diet.

			Fold Change	
Functional classification	Gene name	Symbol	APB	PPB
**Cell growth and development**				
Cell adhesion	Coxsackie virus and adenovirus receptor	CXADR	−2.69	
Cytoskeleton organization	Erythrocyte surface protein band 4.1	EPB41	−2.25	
Cell cycle	DIP13 beta	APPL2	−2.40	
**Cellular metabolism**				
ATP synthesis	ATPase II	ATP8A1	−4.22	
Glycogen metabolism	Glycogen synthase kinase-3 beta (GSK-3 beta)	GSK3B	−2.26	
AA metabolism	Asparagine synthetase domain containing 1	ASNSD1		−2.00
Tryptophan metabolism (catabolism)	Kynurenine 3-monooxygenase	KMO		−2.48
Cholesterol homeostasis	Niemann-Pick disease, type C1	NPC1	−2.05	
Xenobiotic metabolism	UDP glucuronosyltransferase 2 family, polypeptide B15	UGT2B15		−3.30
Aldehyde metabolism	Aldehyde dehydrogenase 1 family, member A1	ALDH1A1		−2.29
**Cell signaling and signal transduction**				
TGF-β signaling pathway	Follistatin	FST	−5.39	−3.73
TGF-β signaling pathway	Thrombospondin 1 precursor	THBS1	−4.28	
Androgen receptor signaling pathway	Nuclear receptor coactivator 2	NCOA2	−4.08	−3.00
Phosphatidylinositol signaling pathway	Inositol polyphosphate 1-phosphatase (IPPase) (IPP)	INPP1	−3.05	
Leukemia inhibitory factor signaling pathway	Leukemia inhibitory factor receptor alpha	LIFR	−2.39	

**Table 5:** Down-regulated cellular trafficking and protein processing-, immune and stress response-, and transcription-translation-associated genes in hepatic tissue of senior vs. young adult dogs fed an animal protein-based (APB) or plant protein-based (PPB) diet.

			Fold Change	
Functional classification	Gene name	Symbol	APB	PPB
**Cellular trafficking and protein processing**				
Endoplasmic reticulum organization	Atlastin GTPase	ATL2		−2.92
Transport	Golgi phosphoprotein 4	GOLIM4	−3.63	
Protein transport	Solute carrier family 15 (H+/peptide transporter)	SLC15A2	−3.22	
Protein transport	Regulating synaptic membrane exocytosis protein 2	RIMS2	−3.03	
Proteolysis	Nardilysin precursor (N-arginine dibasic convertase)	NRD1	−2.13	
Ion transport	Six transmembrane epithelial antigen of the prostate 2	STEAP2		−2.67
**Immune and stress response**				
Immune response	Galectin 8	LGALS8	−4.56	
Immune response	IgA heavy chain constant region	IGHAC	−4.24	
**Transcription-translation**				
Transcription	CG32045-PB, isoform B	FRY	−5.06	
Translation	Eukaryotic translation initiation factor 4E type 3	LOC611215	−3.01	
Histone acetylation	Enhancer of polycomb homolog 1 isoform 2	EPC1	−2.45	
**Miscellaneous and unknown**				
Unknown	Spermatogenesis associated, serine-rich 2	SPATS2	−2.13	
Unknown	CG7020-PA	DIP2B	−2.19	

**Table 6:** Up-regulated genes in hepatic tissue of senior and young adult dogs fed an animal protein-based (APB) vs. plant protein-based (PPB) diet.

			Fold Change	
Functional classification	Gene name	Symbol	Senior	Young
**Cell growth and development**				
Cytoskeleton organization	PDZ and LIM domain 3	PDLIM3	3.08	
Cytoskeleton organization	Erythrocyte surface protein band 4.1	EPB41		2.07
**Cellular metabolism**				
Tetrahydrofolate metabolism	Pipecolic acid oxidase	PIPOX		2.10
Aldehyde metabolism	Aldehyde dehydrogenase 1 family, member A1	ALDH1A1	2.18	
**Cell signaling and signal transduction**				
G-protein mediated signal pathway	Regulator of G-protein signaling 10 (RGS10)	RGS10		2.39
Leukemia inhibitory factor signaling pathway	Leukemia inhibitory factor receptor alpha	LIFR		2.06
**Cellular trafficking and protein processing**				
Protein transport	Golgi phosphoprotein 4	GOLIM4		3.47
Protein transport	Regulating synaptic membrane exocytosis protein 2	RIMS2		2.52
**Miscellaneous and unknown**				
Unknown	CG7020-PA	DIP2B		2.15

**Table 7:** Down-regulated cell growth and development-, cellular metabolism-, and immune and stress response-associated genes in hepatic tissue of senior and young adult dogs fed an animal protein-based (APB) vs. plant protein-based (PPB) diet.

			Fold Change	
Functional classification	Gene name	Symbol	Senior	Young
**Cell growth and development**				
Tumor suppressor	Major facilitator superfamily domain containing 2A	MFSD2A	−2.53	
Autophagy	Autophagy protein 12-like (APG12-like) (ATG12)	ATG12		−4.73
Cell cycle	Cell cycle associated protein 1	CAPRIN1		−4.17
Cell cycle	Retinoblastoma binding protein 4	RBBP4		−3.74
Cell death	TAR DNA binding protein isoform 4	TARDBP		−2.22
**Cellular metabolism**				
ATP synthesis	ATP synthase gamma chain, mitochondrial	ATP5C1		−4.73
AA metabolism	Branched-chain alpha-keto acid dehydrogenase E1 component beta chain	BCKDHB		−3.72
CHO metabolism	Pyruvate dehydrogenase protein X component, mitochondrial precursor	PDHX		−2.38
Lipid metabolism	N-acylsphingosine amidohydrolase (acid ceramidase) 1	ASAH1		−10.63
Glucocorticoid metabolism	Hydroxysteroid (11-beta) dehydrogenase 1	HSD11B1		−4.69
O-linked glycosylation	GalNAc transferase 13	GalNAc-T13		−2.05
Xenobiotic metabolism	UDP-glucuronosyltransferase 2A1 precursor, microsomal	UGT2A1		−4.15
**Immune and stress response**				
Immune response	Ectonucleotide pyrophosphatase/phosphodiesterase 2	ENPP2		−2.82
Oxidative stress	Superoxide dismutase [Mn], mitochondrial precursor	SOD2		−2.22
Oxidative stress	Catalase	CAT		−2.22

**Table 8:** Down-regulated cell signaling and signal transduction-, cellular trafficking and protein processing-, and transcription-translation-associated genes in hepatic tissue of young adult dogs fed an animal protein-based (APB) vs. plant protein-based (PPB) diet.

Functional classification	Gene name	Symbol	Fold Change
**Cell signaling and signal transduction**			
Neurotropin signaling pathway	Mitochondrial import stimulation factor L subunit	YWHAE	−16.21
Hepatocyte growth factor receptor signaling pathway	Met proto-oncogene (hepatocyte growth factor receptor)	MET	−3.33
Wnt signaling pathway	Ras-like protein TC25	RAC1	−3.65
Wnt signaling pathway	Calcineurin A2	PPP3CB	−2.42
Wnt signaling pathway	HMG-box transcription factor 1	HBP1	−2.16
**Cellular trafficking and protein processing**			
Transport	Synaptophysin-like 1 isoform b	SYPL1	−2.26
Protein secretion	Protein disulfide-isomerase A4	PDIA4	−5.35
Protein transport	Ras-related protein Rab-18	RAB18	−4.37
Protein binding	Multiple PDZ domain protein	MPDZ	−3.10
Protein binding	TIP41, TOR signaling pathway regulator-like	TIPRL	−2.55
**Transcription-translation**			
Transcription	Mediator complex subunit 28	MED28	−4.72
Telomere maintenance	Telomeric repeat binding factor 2 interacting protein 1	TERF2IP	−3.26

For validation of microarray data, 5 genes (WFDC2, PFKP, FADS3, GBGT1, and NCOA2) identified to be differentially expressed by age in microarray analysis were selected and validated by quantitative real-time PCR (qRT-PCR) according to methods described previously [9]. Although the magnitude of fold change by microarray vs. qRT-PCR was variable, the direction in gene expression change was identical between the 2 methods (data not shown).

Hepatic lipid composition is presented in Table 9. Senior dogs had greater (P<0.01) concentrations of total lipids and total monounsaturated fatty acids and had a tendency for greater concentrations of total saturated fatty acids (P = 0.09) and total polyunsaturated fatty acids (P = 0.06) as compared to young adult dogs. Despite differences in diet composition and diet-associated blood cholesterol changes as observed in our previous experiment [4], however, diet did not alter hepatic lipid composition in either senior or young adult dogs. No significant interaction between age and diet was observed for hepatic lipid composition.

**Table 9:** Hepatic lipid composition in senior vs. young adult dogs fed an animal protein-based (APB) or plant protein-based (PPB) diet^1^.

	Senior dogs		Young dogs			P – value^2^	
Lipid compositions	APB	PPB	APB	PPB	SEM	Age	Diet
Total lipids	178.2	157.8	120.6	114.7	10.80	<0.01	0.26
Total SAT	100.5	94.9	85.3	81.2	7.38	0.09	0.53
Total MUFA	64.7	52.7	31.5	29.0	5.76	<0.01	0.24
Total PUFA	13.0	10.1	3.8	4.5	3.40	0.06	0.76

1 Values for lipid concentrations are presented as mg/g DM of tissue (n = 3 per treatment).

2 No age×diet interactions were significant (P>0.05).

## Discussion

### Global alterations in gene expression due to age and diet

Of the 13,778 genes expressed in liver tissue, 1.7% (234/13,778) of gene transcripts were differentially expressed by age, while 1.0% (137/13,778) of gene transcripts were altered by diet in this experiment. This observation that a relatively small number of genes was altered by age and diet in these dogs is in agreement with our previous microarray data for cerebral cortex [6], abdominal adipose [7], and skeletal muscle [8] tissues of the same dogs. Therefore, it may be implicated that physiological alteration in the liver due to age and diet, as reported in other body tissues, is likely achieved by a small number of genes and their transcriptional alterations [10]. Age*diet interactions appeared to be present because age-associated gene expression changes in the liver were more common in dogs fed APB (38 genes) than for dogs fed PPB (21 genes). We speculate that because APB had a greater concentration of protein and lipid than PPB, it put more pressure on the liver to metabolize dietary protein and lipids.

### Age-associated alterations in gene expression

The WAP four-disulfide core domain 2 (WFDC2) was greatly up-regulated in senior dogs consuming APB (107.0 fold) or PPB (21.97 fold) in this experiment. The up-regulation of WFDC2 gene has been considered an early biomarker for carcinogenesis, especially for ovarian and pancreatic cancers [11]. It has also been reported that WFDC2 is involved in inflammatory responses and host defense, and its activity is increased in chronically inflamed lungs with cystic fibrosis [12]. Increased expression of genes related to inflammation and immune response was also observed in other tissues from these dogs [6], [7], [8]. In our previous experiment [4], senior dogs fed APB had a greater concentration of blood cholesterol than those fed PPB diet. Increased blood cholesterol concentration has been related to an increased risk of liver inflammation and cystic fibrosis [13]. This may explain why the magnitude of change in WFDC2 gene expression was greater in senior dogs fed APB as compared to those fed PPB. Although physiological significance of WFDC2 in the liver has yet to be identified and dogs used in this experiment were all clinically healthy, it may be worthy to study this gene as a potential biomarker for the progression of liver dysfunction.

Several genes associated with cellular metabolism of amino acids, carbohydrates, lipids, or xenobiotics were affected by age. The up-regulation (2.67 fold) of the D-amino acid oxidase (DAO) gene in senior dogs agrees with previous results of increased DAO activity in the liver of aged rats [14]. This response has been hypothesized to be due to an increased need for detoxification of D-amino acids that may accumulate during aging [14]. The expression of kynurenine 3-monooxygenase (KMO), a key enzyme associated with tryptophan catabolism, was down-regulated (2.48 fold) in senior dogs fed PPB. Age-associated decline in KMO activity was also reported in the rat liver [15]. It is suggested that decreased nicotinic acid synthesis as a result of disturbed tryptophan (kynurenine) catabolism with age may be a reason for age-associated abnormalities (e.g., impaired glucose tolerance) in the liver and other body organs [15].

Genes associated with the glycolytic pathway were differentially expressed in this experiment. Expression of phosphofructokinase (PFKP), which plays a role in the glycolytic flux as the first committed step of glycolysis [16], was up-regulated (4.27 fold) in the liver of senior dogs fed PPB. Moreover, 6-phosphfructo-2-kinase/fructose-2,6-biphosphatase 3 (PFKFB3), which converts fructose-6-phosphate to fructose-2,6-bisphosphate [16], was also up-regulated (3.10 fold) in the liver of senior dogs fed PPB. Fructose-2,6-bisphosphate is a strong allosteric activator of PFKP to increase the rate of glycolysis, whereas it inhibits gluconeogenesis by decreasing activity of fructose-1,6-bisphophatase [16]. Moreover, increased concentrations of intracellular ATP are known to allosterically inhibit activity of PFKP [16]. We observed that the aged liver had a down-regulation (4.22 fold) of ATPase (ATP8A1) gene related to ATP synthesis. Overall, these observations suggest that hepatic glycolytic activity increases but gluconeogenic activity decreases in aged dogs and, therefore, possibly decreased hepatic glucose concentrations. Although it was not measured in this experiment, a reduction in hepatic glucose concentrations has been observed in aged mice [17]. Given the fact that liver is an important regulator of blood glucose concentrations, therefore, our observation in senior dogs suggests that aged liver may have a decreased capacity to maintain blood glucose homeostasis.

The down-regulation (2.26 fold) of glycogen synthase kinase-3 beta gene (GSK3B), which is known to inactivate glycogen synthase [18], in senior dogs fed APB may reflect increased rate of glycogenesis in the aged liver. To our knowledge, however, increased glycogen synthesis or storage in the liver of aged individuals has not been reported, while a decrease in age-related hepatic glycogen storage was observed in aged mice [17]. Apart from its role in glycogen metabolism, it has been reported that hepatic expression of GSK3B declines with age in mice and this reduction is responsible for decreased regenerative ability of aged liver [19]. Therefore, decreased expression of GSK3B observed in this experiment may be more associated with a reduction in regenerative capacity of the liver rather than an increase in hepatic glycogen synthesis in senior dogs.

Senior dogs had greater amounts of hepatic total lipids, saturated fatty acids, and unsaturated fatty acids as compared to young adult dogs. This result was expected because increased hepatic lipid accumulation with age in humans and animals is a well known phenomenon [20], [21]. This response has been frequently associated with an increased risk of age-dependent liver diseases [22]. However, only a small number of genes (FADS3, RDH16, GBGT1, NPC1) involved in hepatic lipid metabolism were differentially expressed by age in this experiment.

Increased expression (4.91 fold) of fatty acid desaturase 3 (FADS3) in the aged liver may indicate the possibility of increased activity of de novo lipogenesis, although the liver is not the major de novo lipogenic organ in dogs [23]. It can be assumed that increased hepatic glycolytic activity observed in this experiment may accelerate the synthesis of citrate, the precursor for fatty acids and cholesterol biosynthesis [24], [25]. As a result, the activity of FADS3, which converts saturated fatty acids as the major end-products of de novo lipogenesis to monounsaturated fatty acids (e.g., oleic acids) as a main storage form in triglycerides [26], is expected to be increased. This may also explain why senior dogs had greater concentrations of hepatic lipids and monounsaturated fatty acids than young adult dogs.

The down-regulation (2.05 fold) of Niemann-Pick disease type C1 (NPC1), as observed in senior dogs consuming APB, may also contribute to increased hepatic lipid concentrations in the aged dogs. The NPC1 gene plays a role in the regulation of intracellular cholesterol trafficking and homeostasis, with its defect leading to an abnormal accumulation of cholesterol and other lipids in hepatic cells [27]. Although hepatic cholesterol concentrations were not measured in this experiment, increased blood cholesterol concentrations were observed in senior dogs [4]. Therefore, age-associated change in NPC1 expression and its effects on hepatic cholesterol concentrations may be a useful measure in future aging studies. Interestingly, Forssman glycolipid synthetase (GBGT1) gene related to Forssman glycolipid biosynthesis was up-regulated in senior dogs consuming APB (3.59 fold) or PPB (4.49 fold). It has been reported that increased expression of GBGT1 reduces the susceptibility to microbial toxins (e.g., Shiga toxins) because Forssman glycolipids, which do not bind microbial toxins, are thought to inhibit the binding of toxin by replacing toxin-binding glycolipid [28]. It is not clear why aged canine liver had increased expression of GBGT1 gene, but deserves attention in future experiments.

In mammals, the liver is the central organ for xenobiotic metabolism. It is well-known that the ability to detoxify xenobiotics in the liver declines with age [1], [29], [30]. This lowered capacity of hepatic xenobiotic clearance is often associated with abnormal drug reactions and further increased risk of liver disease and cancer in the elderly [31]. The UDP glucuronosyltransferase 2B15 (UGT2B15) gene was down-regulated (3.30 fold) in the liver of senior dogs fed PPB. This gene encodes glucuronosyltransferase that catalyzes glucuronidation in phase II reactions of xenobiotic metabolism and, therefore, its mutation increases abnormal drug metabolism and tumorigenesis [32]. Therefore, decreased expression of UGT2B15 in senior dogs may contribute to the decreased efficacy of hepatic xenobiotic metabolism with age. Glutathione-S-transferase pi 1 (GSTP1), which encodes glutathione-S-transferase (GST), was up-regulated in senior dogs fed APB (4.41 fold) or PPB (12.60 fold). The GST gene plays an important role in the clearance of cellular xenobiotics, carcinogens, [33] and defense against oxidative stress [34]. It is likely that increased expression of GSTP1 gene in the liver of aged dogs may reflect a physiological adaptation to an increase in xenobiotic loads and oxidative stress with age. To our knowledge, however, no experiments have reported an age-associated increase in hepatic GSTP1 expression, although it is reported that GSTP1 expression and GST activity in normal colonic mucosa increased with age in female adults [35]. Further research is required to explore age-related GSTP1 regulation on hepatic xenobiotic metabolism and oxidative stress.

There were numerous age-associated alterations in genes related to signaling transduction, such as RAS (RASGRP1), TGF-β (FST, THBS1), androgen receptor (NCOA2), and phosphatidylinositol (INPP1) pathways. Therefore, modification of intracellular signaling pathways may be an integral part of the aging process in the liver. The signaling pathways mentioned above are associated with inflammation and immune response in the liver. RASGRP1 is involved in the development and activation of several immune cell types [36], [37]. Up-regulated RASGRP1 expression (2.63 fold), as observed in senior dogs consuming PPB, may be related to increased inflammatory response as is frequently observed with aging [31]. Senior dogs fed APB (5.39 fold) or PPB (3.73 fold) had decreased expression of follistatin (FST) that has been related to various cellular processes such as cell development, wound healing, apoptosis, and immune response by antagonizing activin activity in the TGF-β signaling pathway [38]. It is suggested that the decreased ability of FST to neutralize activin activity may lead to an increased risk of hepatic pathogenesis such as chronic inflammation and fibrosis [39]. Likewise, the decreased expression (4.28 fold) of thrombospondin 1 (THBS1), as observed in senior dogs fed APB, may also indicate the predisposition of the liver to inflammation and fibrosis with age because THBS1, a mediator of TGF-β signaling pathway, has been implicated in attenuating inflammatory response and fibrosis by limiting angiogenesis in the heart [40]. However, the role of TGF-β signaling pathway in hepatic inflammation and fibrosis remains speculative.

### Diet-associated alterations in gene expression

The liver is the central organ to metabolize dietary nutrients. It has been reported that hepatic gene expression profiles were affected by protein quality and quantity in rats [41]. Moreover, a greater concentration of lipids in the APB diet (22.6%) than in the PPB diet (11.2%) and different fatty acid composition between these 2 diets were expected to induce hepatic gene expression differentially. In this study, however, dietary treatment resulted in a relatively small number of gene expression changes (36 genes) with inconsistent patterns of gene expression (Figure 2). The reason for this observation may be that both diets in this experiment were formulated to contain adequate amounts of dietary protein and essential amino acids for senior or young adult dogs. Furthermore, the lack of effect of dietary treatment on hepatic lipid composition and concentrations in this experiment may also explain why there were the small changes in diet-associated gene expression. Likewise, our previous experiment reported that hematology and blood metabolites involved in liver metabolism were not significantly affected by dietary treatment [4].

The APB diet contained a greater amount of animal-derived lipids high in saturated fatty acids and cholesterols, which are predisposing factors for liver abnormalities in the elderly [42], [43]. Therefore, we hypothesized that APB diet would affect gene expression changes to a greater extent in senior dogs than in young adult dogs. However, of 36 genes differentially expressed by dietary treatment, gene expression changes were more pronounced in young dogs (33 genes) than in senior dogs (3 genes), again suggesting the presence of age*diet interactions. The reason for this observation is not clear, but it is likely due to differences in feeding strategy between young adult and senior dogs. A restricted feeding method was used to maintain body weight of senior dogs in this experiment, which may attenuate the effects of diet on gene expression involved in hepatic metabolism because food restriction has been shown to result in an overall reduction in metabolic rate [10], [44].

Up-regulation of PDLIM3 (3.08 fold) and ALDH1A1 (2.18 fold) genes and down-regulation of MFSD2A gene (2.53 fold) were observed in the liver of senior dogs consuming APB. It is reported that ALDH1A1 expression was positively associated with hepatocyte cytotoxicity in response to saturated fatty acid insults [45]. Therefore, the observation for increased expression of ALDH1A1 gene may indicate that feeding animal-derived ingredient high lipids and saturated fatty acids to senior dogs increases incidence of hepatocyte damage and death. Although MFSD2A gene is highly expressed in the liver [46], its role in liver metabolism has not been elucidated. A recent experiment reported that MFSD2A acts as a tumor suppressor in the lung by regulating expression of genes related to cell cycle and extracellular matrix [47].

Young dogs consuming the APB diet had a down-regulation of several genes associated with cellular metabolism of ATP (ATP5C1), branched chain amino acids (BCKDHB), carbohydrates (PDHX), lipids (ASAH1, HSD11B1), and xenobiotics (UGT2A1). Moreover, genes associated with signal transduction (YWHAE, MET, RAC1, PPP3CB, and HBP1) were also down-regulated in the liver of young dogs consuming APB. The reason for this observation is not clear; however, it may be related to differences in nutrient intake and subsequent nutrient digestion between dogs fed APB and PPB. Based on our calculation using nutrient intake and nutrient digestibility from our previous experiment [4], young dogs fed APB digested 38% greater amount of lipids (32.3 vs. 22.0 g/d for APB vs. PPB) and 37% lower amount of protein (30.4 vs. 44.1 g/d for APB vs. PPB) as compared to young dogs fed PPB. Therefore, the decreased expression (3.72 fold) of BCKDHB, which encodes branched chain α-keto acid dehydrogenase required for branched chain amino acid catabolism, may be a consequence of a lower absorption of branched chain amino acids in young dogs fed APB. Moreover, the decreased gene expression (4.69 fold) of HSD11B1 (11β-hydroxysteroid dehydrogenase type 1) in young dogs fed APB may also be related to high fat intake and absorption. The HSD11B1 is an enzyme that converts 11-dehydrocorticosterone to active corticosterone (cortisol) and is highly expressed in liver and adipose tissue [48]. It has been reported that mice fed a high fat diet had decreased activity of HSD11B1 in the liver and adipose tissue, suggesting that its down-regulation may be an adaptive mechanism in response to high fat intake [48], [49].

The observation for decreased expression of genes associated with antioxidant enzymes, including superoxide dismutase (SOD2) and catalase (CAT) in the liver of young dogs fed APB, was surprising because high lipid and/or cholesterol intake, as observed in young dogs fed APB, is expected to increase hepatic oxidative stress concomitant with increased expression of genes related to antioxidant enzymes [50], [51], [52]. It has been reported that feeding diets containing high cholesterol and lipids to rabbits decreased SOD and CAT activities in the liver [53], [54]. Similar reduction in SOD activity was also observed in the kidney and vascular tissues of rats fed high lipid diets [55]. Taken together, it may be suggested that high lipid and/or cholesterol intake as occurs when consuming animal-derived ingredients, may decrease expression of genes related to antioxidant enzymes and subsequently increase oxidative stress in the liver.

Although statistical differences were detected in expression of several genes due to diet in young dogs, such changes may be of little pathological relevance to hepatic function because all young dogs remained healthy and had normal growth during the entire experiment. In addition, our previous observation for serum metabolites and hematology in young dogs fed APB vs. PPB indicates normal liver function in young adult dogs [4]. It is speculated, therefore, that the diet-associated modulation of hepatic gene expression observed in young dogs may be an adaptive mechanism in response to the distinct diet composition.

In conclusion, using canine microarray technology, we have identified global gene expression in the liver as affected by age and diet. Among transcriptional changes, more genes appeared to be altered by age as compared to diet, but age*diet interactions were also noted. Genes involved in cellular development, metabolism, and signaling transduction were differentially expressed by age and/or diet. In general, the gene expression changes in senior dogs suggest a propensity for liver disease and dysfunction because genes related to inflammation and oxidative stress were up-regulated, whereas genes related to regeneration and xenobiotic metabolism were down-regulated. Diet-induced gene expression changes were likely due to differences in feeding strategy between senior and young adult dogs, and in lipid concentrations between APB and PPB diet. In particular, genes encoding antioxidant enzymes were down-regulated in young adult dogs fed APB. This study, therefore, has highlighted hepatic alterations in global gene expression due to age and diet, providing a useful foundation for future research pertaining to age-dependent changes in hepatic physiology and pathogenesis, and nutritional intervention.

## Materials and Methods

### Animals, diets and experimental design

All experimental procedures were approved by the University of Illinois Institutional Animal Care and Use Committee (IACUC #02056) prior to initiation of the experiment. All animal care, handling, and sampling procedures are detailed in Swanson et al. [4]. In short, 6 senior (average age = 11.1 y at baseline; Kennelwood Inc., Champaign, IL) and 6 young (8 wk at baseline; Marshall Farms USA, Inc., North Rose, NY) female beagles were randomly allotted to 1 of 2 dietary treatments for 12 months. One diet was an animal protein-based diet (APB) containing 28.0% crude protein (CP), 22.6% fat, and 4.8% total dietary fiber (TDF). The other diet was a plant protein-based diet (PPB) containing 25.5% CP, 11.2% fat, and 15.2% TDF. The APB diet was formulated with brewer's rice, chicken by-product meal, and poultry fat, while the PPB diet consisted mainly of corn, soybean meal, and wheat middlings. Specific details of these 2 dietary treatments were reported previously [4]. Both diets were formulated to meet or exceed all nutrient requirements for canine growth and maintenance according to the Association of American Feed Control Officials [56].

Young dogs were fed *ad libitum* to allow for adequate growth, whereas senior dogs were fed a restricted amount of the diet to maintain baseline body weight throughout the experiment. Senior dogs maintained body weight and a fairly constant food intake over the course of the experiment, consuming similar amounts of food (APB: 199.1 vs. 183.5 g dry matter/d; PPB: 250.4 vs. 235.2 g dry matter/d), energy (APB: 1071 vs. 987 kcal/d; PPB: 1190 vs. 1117 kcal/d), protein (APB: 55.7 vs. 51.4 g/d; PPB: 63.9 vs. 60.0 g/d), fat (APB: 45.0 vs. 41.5 g/d; PPB: 28.0 vs. 26.3 g/d), and fiber (APB: 9.6 vs. 8.8 g/d; PPB: 38.1 vs. 35.8 g/d) during the early (3 months after baseline) and late (10 months after baseline) stages of the experiment. Young dogs also had similar food (APB: 150.4 vs. 148.6 g dry matter/d; PPB: 225.6 vs. 237.7 g dry matter/d), energy (APB: 809 vs. 800 kcal/d; PPB: 1071 vs. 1129 kcal/d), protein (APB: 42.1 vs. 41.6 g/d; PPB: 57.7 vs. 60.6 g/d), fat (APB: 34.0 vs. 33.6 g/d; PPB: 25.3 vs. 26.6 g/d), and fiber (APB: 7.2 vs. 7.1 g/d; PPB: 34.3 vs. 36.1 g/d) intakes at the 3 and 10-month time points. Although similar food and macronutrient intakes were observed over time in young dogs, it occurred with much different body weights (6.2 kg at 3 months vs. 9.0 kg at 10 months). Therefore, macronutrient intake per kg body weight was much greater at 3 months, a period of rapid growth, than at 10 months when growth is much slower.

### Sample collection, RNA extraction, and microarray data analyses

After 12 months of experiment, dogs were fasted for 12 hours and euthanized using a lethal dose (130 mg/kg body weight) of sodium pentobarbital (Euthasol®, Virbac Corp., Fort Worth, TX). Liver tissue was immediately collected, flash frozen using liquid nitrogen, and stored at −80°C. Total cellular RNA was isolated from liver tissue using Trizol (Invitrogen, Carlsbad, CA). RNA concentration was measured using a ND-1000 spectrophotometer (Nanodrop Technologies, Wilmington, DE) and RNA integrity was verified on a 1.2% denaturing agarose gel.

The procedures for microarray data analyses were described previously by Swanson et al. [6]. In short, the prepared RNA samples were hybridized to Affymetrix GeneChip® Canine Genome Arrays (Affymetrix, Santa Clara, CA). After hybridization, chips were washed and stained with streptavidin-conjugated phycoerythrin dye (Invitrogen) enhanced with biotinylated goat anti-streptavidin antibody (Vector Laboratories, Burlingame, CA) utilizing an Affymetrix GeneChip® Fluidics Station 450 and GeneChip® Operating Software. Images were then scanned using an Affymetrix GeneChip® Scanner 3000. Of the 23,836 probe sets on the array, 13,778 probe sets were expressed in the liver tissue and were used to determine effects of age and diet on gene expression profiles. Functional classification was made by the database SOURCE (http://source.stanford.edu) [57]. All microarray data have been deposited in the Gene Expression Omnibus (GEO) repository at the National Center for Biotechnology Information (NCBI) archives (http://www.ncbi.nlm.nih.gov/geo).

### Liver lipid analyses

Lipid concentrations in the liver tissue were measured by gas chromatography [58]. In short, the liver tissue was homogenized using a Fisher Powergen Model 125 tissue homogenizer (Fisher Scientific, Hampton, NH). Internal standards and 0.1 g liver tissue were passed through hexane to extract the lipids. Fatty acid composition of the extracted lipids was measured using gas chromatography (Hewlett-Packard 5890A Series II) and external standards for identification and quantification.

### Statistical analysis

Individual animal was the experimental unit for all analyses. Differential expression of the microarray data was evaluated using the limma package [59]. A linear model for the four age x diet groups was fit for each probe set. Differences between groups were then extracted from the model as contrasts. An empirical Bayes “shrinkage” method was employed on the standard errors to improve power for small sample sizes [59]. Lastly, multiple test correction of P-values was done using the false discovery rate (FDR) method [60]. Gene transcripts having >2.0-fold change and FDR <0.10 were considered significantly different. Data for hepatic lipid concentrations were analyzed using the Proc Mixed procedure of SAS (SAS Inst, Inc., Cary, NC). A probability of P<0.05 was accepted statistically significant and 0.05<P<0.10 was considered as a trend for hepatic lipid concentrations.
